# Supplementary material for: Advancing training effectiveness prediction in mass sport through longitudinal data: A mathematical model approach based on the Fitness-Fatigue Model
Source: PLoS One. 2025 Dec 3;20(12):e0337824. doi: 10.1371/journal.pone.0337824 (PMC12674547; doi:10.1371/journal.pone.0337824)
Supplement: S1 Table — (DOCX) [file pone.0337824.s001.docx]

**S1 Table. Physiological interpretation of optimized FFM parameters**

| Parameter | Symbol | Physiological Interpretation |
| --- | --- | --- |
| Adaptation decay rate | *a* | A larger value indicates faster adaptation loss and accounts for individual differences in adaptation |
| Adaptation time constant | *τ_a_* | A larger value indicates slower adaptation changes |
| Adaptation gain coefficient | *K_a_* | A larger value indicates greater adaptation per unit of external load |
| Adaptation initial constant | *C_1_* | Represents the baseline adaptation level before training begins |
| Fatigue decay rate | *f* | A larger value indicates faster fatigue recovery and accounts for individual differences in fatigue |
| Fatigue time constant | *τ_f_* | A larger value indicates slower fatigue changes |
| Fatigue gain coefficient | *K_f_* | A larger value indicates greater fatigue per unit of external load |
| Fatigue initial constant | *C_2_* | Represents the baseline fatigue level before training begins |
