# Supplementary material for: Advancing training effectiveness prediction in mass sport through longitudinal data: A mathematical model approach based on the Fitness-Fatigue Model
Source: PLoS One. 2025 Dec 3;20(12):e0337824. doi: 10.1371/journal.pone.0337824 (PMC12674547; doi:10.1371/journal.pone.0337824)
Supplement: S2 Table — (DOCX) [file pone.0337824.s002.docx]

**S2 Table. Model parameter estimation results (using ΔHRR1 to calculate the output indicators)**

| Subjects number | *a* | *τ_a_* | *K_a_* | *C_1_* | *f* | *τ_f_* | *K_f_* | *C_2_* |
| --- | --- | --- | --- | --- | --- | --- | --- | --- |
| 1 | 2.508 | 0.001796 | 1.539 | -1.143 | 0.1035 | 0.9944 | -0.1092 | 1.431 |
| 2 | -2.555 | 0.1507 | 0.6155 | 0.7542 | -2.061 | 0.1061 | -0.5951 | 0.4354 |
| 3 | -1.254 | 8.397 | -0.6521 | 4.255 | 1.44 | 0.01583 | 0.09708 | 1.485 |
| 4 | -0.2877 | 0.6219 | 1.169 | 0.4987 | 0.1788 | 0.06687 | -0.03572 | 0.2 |
| 5 | 46.37 | 2.816 | 1.578 | 0.2288 | 46.63 | 10.55 | -0.1773 | 0.1397 |
| 6 | 2.694 | 0.2524 | 2.108 | -0.06335 | 1.995 | 0.115 | -0.5629 | 0.8866 |
| 7 | 32.34 | 3.321 | 1.246 | 0.6619 | 32.62 | 10.36 | -1.015 | 0.6065 |
| 8 | 1.123 | 1.818 | 2.122 | -0.2186 | 0.1844 | 1.88 | -0.7875 | 1.012 |
| 9 | 2.92 | 0.4815 | 1.92 | 0.3611 | 2.494 | 0.3385 | -0.7358 | 0.9989 |
| 10 | 1.55 | 1.018 | 1.358 | 0.6527 | 1.485 | 0.7691 | -0.2737 | 0.8694 |
| 11 | 3.919 | 0.03173 | 17.25 | -97.2 | 9.512 | 0.008804 | 15.03 | -102.6 |
| 12 | -0.2233 | 0.4369 | 0.8955 | 0.9981 | 0.1987 | 1.812 | 0.02271 | 0.6634 |
| 13 | 1.764 | 1.042 | 1.85 | 0.6102 | 1.593 | 1.601 | -0.2016 | 0.987 |
