# Supplementary material for: Advancing training effectiveness prediction in mass sport through longitudinal data: A mathematical model approach based on the Fitness-Fatigue Model
Source: PLoS One. 2025 Dec 3;20(12):e0337824. doi: 10.1371/journal.pone.0337824 (PMC12674547; doi:10.1371/journal.pone.0337824)
Supplement: S3 Table — (DOCX) [file pone.0337824.s003.docx]

**S3 Table. Model parameter estimation results (using HRr% to calculate the output indicators)**

| Subjects number | *a* | *τ_a_* | *K_a_* | *C_1_* | *f* | *τ_f_* | *K_f_* | *C_2_* |
| --- | --- | --- | --- | --- | --- | --- | --- | --- |
| 1 | 0.9156 | 0.6328 | 0.8469 | 0.3723 | 1.144 | 0.9035 | -0.201 | 0.253 |
| 2 | 0.2936 | 0.09776 | 0.974 | 0.5519 | 0.6417 | 6.541 | -0.00208 | 0.3009 |
| 3 | 1.054 | 0.1076 | 1.048 | 0.8858 | 1.429 | 0.07285 | 0.05137 | 0.3627 |
| 4 | 0.6357 | 0.3183 | 1.253 | 0.08454 | 0.8249 | 0.4075 | -0.3203 | 0.1328 |
| 5 | -2.572 | 0.09956 | 1.924 | -0.3657 | -3.702 | 0.1316 | -1.076 | 0.7727 |
| 6 | 0.5683 | 0.1204 | 2.57 | -0.3185 | -1.041 | 0.8985 | -1.227 | 1.622 |
| 7 | 1.492 | 0.976 | 0.7205 | 0.6088 | 1.671 | 2.757 | -0.6315 | 0.5659 |
| 8 | -4.493 | 0.1333 | 1.319 | -0.04961 | -4.606 | 0.09645 | -0.3861 | 0.267 |
| 9 | 3.871 | 0.06524 | 1.019 | 0.05604 | 3.763 | 0.09531 | -0.7409 | 0.4661 |
| 10 | 10.96 | 0.4374 | 1.095 | 0.08467 | 10.77 | 0.6052 | -0.8847 | 0.6045 |
| 11 | 2.546 | 1.816 | 3.583 | -16.2 | -0.8965 | 1.81 | -2.997 | -12.11 |
| 12 | -1.525 | 1.154 | 0.9811 | 0.9646 | -1.417 | 0.2358 | -0.3361 | 0.9935 |
| 13 | 0.8411 | 0.2113 | 1.347 | 0.06718 | 0.9342 | 8.712 | -0.4146 | 0.1502 |
