# Supplementary material for: Advancing training effectiveness prediction in mass sport through longitudinal data: A mathematical model approach based on the Fitness-Fatigue Model
Source: PLoS One. 2025 Dec 3;20(12):e0337824. doi: 10.1371/journal.pone.0337824 (PMC12674547; doi:10.1371/journal.pone.0337824)
Supplement: S4 Table — (DOCX) [file pone.0337824.s004.docx]

**S4 Table. Model parameter estimation results (using TL_HRV_ to calculate the output indicators)**

| Subjects number | *a* | *τ_a_* | *K_a_* | *C_1_* | *f* | *τ_f_* | *K_f_* | *C_2_* |
| --- | --- | --- | --- | --- | --- | --- | --- | --- |
| 1 | 2.331 | 3.398 | 1.792 | 0.2014 | 2.356 | 5.739 | -0.5023 | 0.4064 |
| 2 | 0.4986 | 0.6342 | 1.361 | 0.4275 | 0.7781 | 0.7915 | -0.4706 | 0.3312 |
| 3 | 2.977 | 0.1937 | 2.149 | 0.08766 | 3.549 | 0.96 | -0.7762 | -0.1828 |
| 4 | 0.8399 | 2.515 | 1.33 | 0.4857 | 1.15 | 3.314 | 0.004622 | 0.3077 |
| 5 | 5.227 | 0.5736 | 3.564 | -0.9231 | 7.508 | 1.076 | 3.836 | -11.53 |
| 6 | -0.3712 | 0.9018 | 1.456 | 0.8874 | 1.452 | 0.899 | -0.446 | -0.7036 |
| 7 | -11.88 | 3.218 | 1.161 | 0.4728 | -11.81 | 8.235 | -0.5857 | 0.5739 |
| 8 | -1.418 | 1.355 | 1.063 | -0.07162 | 0.5466 | -0.00373 | 0.621 | -1.993 |
| 9 | 0.9267 | 0.226 | 1.268 | 0.5099 | 1.399 | 0.1585 | 0.1249 | 0.13 |
| 10 | 0.2924 | 0.1859 | 0.8962 | 0.5635 | 0.336 | 0.08985 | -0.5494 | 0.661 |
| 11 | 1.345 | 0.8528 | 4.439 | 2.691 | 0.4257 | 1.127 | 0.9047 | 3.966 |
| 12 | -18.93 | 7.429 | 1.541 | 0.6433 | -19.11 | 1.139 | -0.4643 | 1.025 |
| 13 | 3.535 | 0.5419 | 1.201 | 0.5897 | 3.662 | 0.7946 | -0.3052 | 0.6547 |
