# Supplementary material for: Advancing training effectiveness prediction in mass sport through longitudinal data: A mathematical model approach based on the Fitness-Fatigue Model
Source: PLoS One. 2025 Dec 3;20(12):e0337824. doi: 10.1371/journal.pone.0337824 (PMC12674547; doi:10.1371/journal.pone.0337824)
Supplement: S5 Table — (DOCX) [file pone.0337824.s005.docx]

**S5 Table. Evaluation results of model fitting effect (using ΔHRR1 to calculate the output indicators)**

| Subjects number | the optimized model | | | the original model | | |
| --- | --- | --- | --- | --- | --- | --- |
|  | SSE | RMSE | R^2^ | SSE | RMSE | R^2^ |
| 1 | 0.2048 | 0.1067 | 0.8414 | 0.3152 | 0.1171 | 0.7558 |
| 2 | 1.1470 | 0.2100 | 0.6211 | 1.4695 | 0.2177 | 0.5146 |
| 3 | 0.8757 | 0.2206 | 0.7436 | 1.6996 | 0.2718 | 0.5023 |
| 4 | 0.8514 | 0.2175 | 0.7216 | 0.9339 | 0.2015 | 0.6946 |
| 5 | 2.7939 | 0.3738 | 0.7881 | 2.8043 | 0.3418 | 0.4826 |
| 6 | 1.1992 | 0.2738 | 0.7017 | 1.7525 | 0.2889 | 0.564 |
| 7 | 4.4293 | 0.4961 | 0.5844 | 5.8023 | 0.5023 | 0.4556 |
| 8 | 1.7115 | 0.3001 | 0.5968 | 1.8479 | 0.2775 | 0.5646 |
| 9 | 2.7831 | 0.4307 | 0.5375 | 2.9629 | 0.3849 | 0.5076 |
| 10 | 0.6914 | 0.2017 | 0.7487 | 0.7161 | 0.1804 | 0.7397 |
| 11 | 3.4821 | 0.4398 | 0.6023 | 5.7006 | 0.4978 | 0.349 |
| 12 | 0.5288 | 0.1668 | 0.6978 | 0.6043 | 0.1587 | 0.6546 |
| 13 | 1.0327 | 0.2395 | 0.6452 | 1.0898 | 0.2177 | 0.6256 |
