# Supplementary material for: Advancing training effectiveness prediction in mass sport through longitudinal data: A mathematical model approach based on the Fitness-Fatigue Model
Source: PLoS One. 2025 Dec 3;20(12):e0337824. doi: 10.1371/journal.pone.0337824 (PMC12674547; doi:10.1371/journal.pone.0337824)
Supplement: S6 Table — (DOCX) [file pone.0337824.s006.docx]

**S6 Table Evaluation results of model fitting effect (using HRr% to calculate the output indicators)**

| Subjects number | the optimized model | | | the original model | | |
| --- | --- | --- | --- | --- | --- | --- |
|  | SSE | RMSE | R^2^ | SSE | RMSE | R^2^ |
| 1 | 0.2043 | 0.1065 | 0.7889 | 0.2223 | 0.0983 | 0.7703 |
| 2 | 0.9316 | 0.1930 | 0.4961 | 1.1972 | 0.1998 | 0.3524 |
| 3 | 1.0932 | 0.2464 | 0.6296 | 1.3176 | 0.2393 | 0.5536 |
| 4 | 1.2582 | 0.2644 | 0.6312 | 1.2752 | 0.2355 | 0.6262 |
| 5 | 2.1997 | 0.3403 | 0.6199 | 1.7996 | 0.2797 | 0.5086 |
| 6 | 3.4190 | 0.4774 | 0.4722 | 3.9290 | 0.4434 | 0.3929 |
| 7 | 1.0109 | 0.2514 | 0.6583 | 6.3007 | 0.5234 | 0.1425 |
| 8 | 1.5756 | 0.2880 | 0.7043 | 2.4921 | 0.3222 | 0.5324 |
| 9 | 1.7629 | 0.3428 | 0.6099 | 2.4477 | 0.3498 | 0.4584 |
| 10 | 1.3759 | 0.2845 | 0.8071 | 1.1465 | 0.2337 | 0.5750 |
| 11 | 14.6552 | 0.9023 | 0.3162 | 15.8923 | 0.8312 | 0.2585 |
| 12 | 0.8278 | 0.2087 | 0.7268 | 1.6346 | 0.2610 | 0.4605 |
| 13 | 2.3517 | 0.3615 | 0.4277 | 3.0025 | 0.3613 | 0.2693 |
