# Supplementary material for: Advancing training effectiveness prediction in mass sport through longitudinal data: A mathematical model approach based on the Fitness-Fatigue Model
Source: PLoS One. 2025 Dec 3;20(12):e0337824. doi: 10.1371/journal.pone.0337824 (PMC12674547; doi:10.1371/journal.pone.0337824)
Supplement: S7 Table — (DOCX) [file pone.0337824.s007.docx]

**S7 Table. Evaluation results of model fitting effect (using TL_HRV_ to calculate the output indicators)**

| Subjects number | the optimized model | | | the original model | | |
| --- | --- | --- | --- | --- | --- | --- |
|  | SSE | RMSE | R^2^ | SSE | RMSE | R^2^ |
| 1 | 1.3495 | 0.2904 | 0.6028 | 2.7267 | 0.3521 | 0.4468 |
| 2 | 0.2795 | 0.1079 | 0.8764 | 0.3100 | 0.1034 | 0.8629 |
| 3 | 10.0397 | 0.7468 | 0.4560 | 14.5642 | 0.7958 | 0.2109 |
| 4 | 0.3865 | 0.1508 | 0.8275 | 0.4161 | 0.1375 | 0.8143 |
| 5 | 7.3860 | 0.6235 | 0.4030 | 12.0086 | 0.6931 | 0.2523 |
| 6 | 10.3171 | 0.8293 | 0.5399 | 10.7287 | 0.7324 | 0.5216 |
| 7 | 1.7894 | 0.3244 | 0.6060 | 2.0042 | 0.3018 | 0.5587 |
| 8 | 2.4808 | 0.3712 | 0.5392 | 2.7062 | 0.3430 | 0.4973 |
| 9 | 1.3786 | 0.3138 | 0.6096 | 1.7414 | 0.3027 | 0.5069 |
| 10 | 0.0893 | 0.0747 | 0.9497 | 0.1202 | 0.0757 | 0.9322 |
| 11 | 1.7546 | 0.3213 | 0.7186 | 1.8933 | 0.2934 | 0.6963 |
| 12 | 2.1344 | 0.3352 | 0.9454 | 2.1535 | 0.3060 | 0.5175 |
| 13 | 1.2547 | 0.2640 | 0.5232 | 1.6005 | 0.2638 | 0.3918 |
