# Supplementary material for: Advancing training effectiveness prediction in mass sport through longitudinal data: A mathematical model approach based on the Fitness-Fatigue Model
Source: PLoS One. 2025 Dec 3;20(12):e0337824. doi: 10.1371/journal.pone.0337824 (PMC12674547; doi:10.1371/journal.pone.0337824)
Supplement: S8 Table — (DOCX) [file pone.0337824.s008.docx]

**S8 Table. Evaluation results of model prediction ability and** **temporal dependency analysis (using ΔHRR1 to calculate the output indicators)**

| Subjects number | the optimized model | | | | the original model | | | |
| --- | --- | --- | --- | --- | --- | --- | --- | --- |
|  | MAPE (%) | RMSE | ρ | *P*-value | MAPE (%) | RMSE | ρ | *P*-value |
| 1 | 19.27 | 0.2529 | -0.314 | 0.564 | 22.73 | 0.2310 | -0.886 | 0.033 |
| 2 | 11.72 | 0.2199 | -0.450 | 0.230 | 8.35 | 0.1646 | -0.133 | 0.744 |
| 3 | 30.83 | 0.6604 | -0.464 | 0.302 | 34.57 | 0.6790 | -0.321 | 0.498 |
| 4 | 8.93 | 0.1479 | 0.536 | 0.236 | 9.37 | 0.1545 | 0.571 | 0.200 |
| 5 | 19.39 | 0.3532 | -0.536 | 0.236 | 19.56 | 0.3582 | 0.250 | 0.595 |
| 6 | 23.90 | 0.4747 | 0.257 | 0.658 | 22.11 | 0.4800 | -0.029 | 1.000 |
| 7 | 28.60 | 0.5107 | -0.036 | 0.964 | 30.09 | 0.5366 | 0.000 | 1.000 |
| 8 | 17.39 | 0.2642 | 0.607 | 0.167 | 9.10 | 0.154 | 0.429 | 0.354 |
| 9 | 9.77 | 0.2345 | -0.486 | 0.356 | 10.67 | 0.2634 | -0.314 | 0.564 |
| 10 | 30.32 | 0.8135 | 0.029 | 1.000 | 31.11 | 0.8252 | 0.257 | 0.658 |
| 11 | 47.31 | 0.7876 | -0.321 | 0.498 | 20.17 | 0.4458 | 0.464 | 0.302 |
| 12 | 10.44 | 0.1276 | 0.000 | 1.000 | 10.73 | 0.1277 | -0.036 | 0.964 |
| 13 | 13.64 | 0.1920 | -0.071 | 0.906 | 11.26 | 0.1623 | -0.143 | 0.783 |

Note: ρ represents Spearman correlation coefficient between prediction horizon and absolute percentage error; *P*-value represents significance of time dependency.
