# Supplementary material for: Advancing training effectiveness prediction in mass sport through longitudinal data: A mathematical model approach based on the Fitness-Fatigue Model
Source: PLoS One. 2025 Dec 3;20(12):e0337824. doi: 10.1371/journal.pone.0337824 (PMC12674547; doi:10.1371/journal.pone.0337824)
Supplement: S9 Table — (DOCX) [file pone.0337824.s009.docx]

**S9 Table. The predicted values and actual values obtained from the model (using ΔHRR1 to calculate the output indicators and taking Subject 4 as an example)**

| Subjects number | Actual data | Predictive data | Predicted difference | |
| --- | --- | --- | --- | --- |
| 4 | 1.360641 | 1.356902 | | -0.00374 |
|  | 1.555493 | 1.443383 | | -0.11211 |
|  | 1.445115 | 1.418265 | | -0.02685 |
|  | 1.369648 | 1.244959 | | -0.12469 |
|  | 1.079425 | 1.363802 | | 0.284377 |
|  | 1.561999 | 1.368205 | | -0.19379 |
|  | 1.446003 | 1.368915 | | -0.07709 |
