# Supplementary material for: Advancing training effectiveness prediction in mass sport through longitudinal data: A mathematical model approach based on the Fitness-Fatigue Model
Source: PLoS One. 2025 Dec 3;20(12):e0337824. doi: 10.1371/journal.pone.0337824 (PMC12674547; doi:10.1371/journal.pone.0337824)
Supplement: S10 Table — (DOCX) [file pone.0337824.s010.docx]

**S10 Table. Evaluation results of model prediction ability and** **temporal dependency analysis (using HRr% to calculate the output indicators)**

| Subjects number | the optimized model | | | | the original model | | | |
| --- | --- | --- | --- | --- | --- | --- | --- | --- |
|  | MAPE (%) | RMSE | ρ | *P*-value | MAPE (%) | RMSE | ρ | *P*-value |
| 1 | 23.27 | 0.3854 | -0.029 | 1.000 | 22.92 | 0.3615 | -0.657 | 0.175 |
| 2 | 17.11 | 0.3321 | 0.083 | 0.843 | 15.58 | 0.2694 | -0.017 | 0.982 |
| 3 | 21.43 | 0.4495 | -0.393 | 0.396 | 21.54 | 0.4583 | -0.250 | 0.595 |
| 4 | 13.90 | 0.2709 | 0.536 | 0.236 | 13.86 | 0.2637 | 0.536 | 0.236 |
| 5 | 16.26 | 0.2871 | 0.286 | 0.556 | 19.70 | 0.3392 | 0.464 | 0.302 |
| 6 | 31.64 | 0.4959 | 0.429 | 0.419 | 14.26 | 0.2671 | -0.771 | 0.103 |
| 7 | 21.76 | 0.326 | -0.786 | 0.048 | 23.75 | 0.3274 | -0.536 | 0.236 |
| 8 | 16.26 | 0.2647 | 0.679 | 0.110 | 14.47 | 0.1934 | 0.357 | 0.444 |
| 9 | 19.20 | 0.4051 | -0.314 | 0.564 | 15.07 | 0.2593 | -0.714 | 0.136 |
| 10 | 32.93 | 1.1722 | 0.200 | 0.714 | 33.80 | 1.1627 | 0.200 | 0.714 |
| 11 | 25.84 | 0.6603 | 0.571 | 0.200 | 30.62 | 0.6566 | 0.786 | 0.048 |
| 12 | 15.36 | 0.2300 | 0.143 | 0.783 | 8.83 | 0.1574 | 0.143 | 0.783 |
| 13 | 12.31 | 0.2233 | -0.071 | 0.906 | 12.38 | 0.2023 | -0.107 | 0.840 |

Note: ρ represents Spearman correlation coefficient between prediction horizon and absolute percentage error; *P*-value represents significance of time dependency.
