# Supplementary material for: Advancing training effectiveness prediction in mass sport through longitudinal data: A mathematical model approach based on the Fitness-Fatigue Model
Source: PLoS One. 2025 Dec 3;20(12):e0337824. doi: 10.1371/journal.pone.0337824 (PMC12674547; doi:10.1371/journal.pone.0337824)
Supplement: S11 Table — (DOCX) [file pone.0337824.s011.docx]

**S11 Table. The predicted values and actual values obtained from the model (using HRr% to calculate the output indicators and taking Subject 4 as an example)**

| Subjects number | Actual data | Predictive data | Predicted difference |
| --- | --- | --- | --- |
| 4 | 1.451976 | 1.337086 | -0.11489 |
|  | 1.786395 | 1.449783 | -0.33661 |
|  | 1.247288 | 1.416527 | 0.16924 |
|  | 1.217008 | 1.18998 | -0.02703 |
|  | 1.242096 | 1.345002 | 0.102906 |
|  | 1.827955 | 1.350407 | -0.47755 |
|  | 1.69623 | 1.350897 | -0.34533 |
