# Supplementary material for: Advancing training effectiveness prediction in mass sport through longitudinal data: A mathematical model approach based on the Fitness-Fatigue Model
Source: PLoS One. 2025 Dec 3;20(12):e0337824. doi: 10.1371/journal.pone.0337824 (PMC12674547; doi:10.1371/journal.pone.0337824)
Supplement: S12 Table — (DOCX) [file pone.0337824.s012.docx]

**S12 Table Evaluation results of model prediction ability and** **temporal dependency analysis (using TL_HRV_ to calculate the output indicators)**

| Subjects number | the optimized model | | | | the original model | | | |
| --- | --- | --- | --- | --- | --- | --- | --- | --- |
|  | MAPE (%) | RMSE | ρ | *P*-value | MAPE (%) | RMSE | ρ | *P*-value |
| 1 | 6.67 | 0.1452 | -0.257 | 0.658 | 8.78 | 0.1513 | -0.600 | 0.242 |
| 2 | 16.65 | 0.3055 | 0.800 | 0.014 | 17.68 | 0.3308 | 0.533 | 0.148 |
| 3 | 82.51 | 1.3039 | 0.357 | 0.444 | 85.33 | 1.3437 | 0.500 | 0.267 |
| 4 | 3.99 | 0.0825 | -0.071 | 0.906 | 4.92 | 0.0849 | -0.607 | 0.167 |
| 5 | 19.88 | 0.7489 | 0.000 | 1.000 | 24.37 | 0.7797 | 0.071 | 0.906 |
| 6 | 86.57 | 1.406 | -0.943 | 0.017 | 76.66 | 1.4232 | -0.771 | 0.103 |
| 7 | 19.33 | 0.2749 | 0.107 | 0.840 | 18.8 | 0.2661 | 0.107 | 0.840 |
| 8 | 32.53 | 0.4523 | -0.071 | 0.906 | 27.19 | 0.4071 | 0.036 | 0.964 |
| 9 | 32.91 | 0.6277 | 0.086 | 0.919 | 94.78 | 0.6197 | 0.086 | 0.919 |
| 10 | 4.27 | 0.0638 | 0.143 | 0.803 | 3.31 | 0.0485 | -0.029 | 1.000 |
| 11 | 31.99 | 0.5088 | 0.286 | 0.556 | 32.78 | 0.5213 | 0.500 | 0.267 |
| 12 | 18.48 | 0.3627 | 0.429 | 0.354 | 18.41 | 0.3609 | 0.429 | 0.354 |
| 13 | 9.87 | 0.1472 | -0.143 | 0.783 | 10.4 | 0.1556 | -0.429 | 0.354 |

Note: ρ represents Spearman correlation coefficient between prediction horizon and absolute percentage error; *P*-value represents significance of time dependency.
