# Supplementary material for: Advancing training effectiveness prediction in mass sport through longitudinal data: A mathematical model approach based on the Fitness-Fatigue Model
Source: PLoS One. 2025 Dec 3;20(12):e0337824. doi: 10.1371/journal.pone.0337824 (PMC12674547; doi:10.1371/journal.pone.0337824)
Supplement: S13 Table — (DOCX) [file pone.0337824.s013.docx]

**S13 Table. The predicted values and actual values obtained from the model (using TL_HRV_ to calculate the output indicators and taking Subject 4 as an example)**

| Subjects number | Actual data | Predictive data | Predicted difference | |
| --- | --- | --- | --- | --- |
| 4 | 1.543528 | 1.345098 | | -0.19843 |
|  | 1.406519 | 1.439944 | | 0.033424 |
|  | 1.400882 | 1.411956 | | 0.011073 |
|  | 1.270257 | 1.221293 | | -0.04896 |
|  | 1.336976 | 1.351759 | | 0.014784 |
|  | 1.303108 | 1.356309 | | 0.053201 |
|  | 1.39643 | 1.356721 | | -0.03971 |
