# Supplementary material for: Advancing training effectiveness prediction in mass sport through longitudinal data: A mathematical model approach based on the Fitness-Fatigue Model
Source: PLoS One. 2025 Dec 3;20(12):e0337824. doi: 10.1371/journal.pone.0337824 (PMC12674547; doi:10.1371/journal.pone.0337824)
Supplement: Attachment — Submitted filename: Response to Reviewers.docx [file pone.0337824.s016.docx]

Dear Editors and Reviewers,

We sincerely appreciate the thorough review process conducted by ***PLOS ONE*** for our manuscript titled **“Advancing training effectiveness prediction in mass sport through longitudinal data: a mathematical model approach based on the Fitness-Fatigue Model”**. We have meticulously examined all comments from the two reviewers and have implemented detailed revisions accordingly. Furthermore, we have addressed the journal requirements from the editorial office, including uploading all data underlying the findings described in our manuscript to the supporting information as per the journal's policy.

Given the length of this response, we have presented the reviewers' comments in blue font and our responses in black font to ensure clarity and ease of distinction.

Once again, we thank the editorial team and reviewers for their hard work. Below, please find our detailed point-by-point responses to the reviewers' comments and the journal requirements.

# To Reviewer 1:

General response

Thank you for your positive feedback and valuable suggestions. Your insightful comments have been a great encouragement and have guided us to enhance the quality of our manuscript. We have thoroughly addressed each of your suggestions and made comprehensive revisions to enhance the clarity and overall quality of our work.

The detailed modifications are outlined in the Point-by-point responses below.

Point-by-point responses

**Abstract**

Comment 1

The article presents a meticulously developed mathematical approach to optimize the Fitness-Fatigue Model (FFM) in the context of mass sports. The most unique aspects of the study are the model's ability to capture individual differences, to make personalized predictions using longitudinal data, and its potential for integration with wearable technologies. The main outline of the study is clearly stated in the abstract.

The abstract should state the research question more clearly and briefly touch on the limitations of the study. The rationale for choosing the heart rate variability (HRV) and heart rate recovery (HRR) indicators can be emphasized in the introduction of the abstract. In addition, the presentation of quantitative results (R^2^, RMSE, MAPE values) as prominent findings in the abstract will strengthen the article.

Response:

Thank you for the positive feedback on our article and the insightful comments regarding the abstract. We have carried out thorough revisions to the abstract in accordance with your recommendations.

Firstly, we have included a brief summary of the research background, highlighting the need for scientific training load assessment in mass sports and pointing out the current limitations of the Fitness-Fatigue Model. This addition is intended to clarify the research question.

As per your suggestion, we have also briefly addressed the limitations of the study and provided an outlook on future research directions in the end of the abstract.

In addition, to better explain why we chose heart rate variability and heart rate recovery indicators, we have used the term “wearable-compatible” in the abstract. This term is in line with our study's goal of identifying generalizable model output indicators.

Lastly, we have enhanced the abstract by adding a detailed account of the quantitative results, including R², RMSE, and MAPE values, which we believe strengthens the persuasiveness of the article.

Below is the revised abstract:

“Despite the critical need for scientific training load assessment in mass sports, the Fitness-Fatigue Model (FFM) requires further mathematical optimization and practical output indicators. The aim of this study was to optimize the mathematical relationship between "adaptation" and "fatigue" in the FFM, identify generalizable model output indicators, and evaluate its performance in predicting training effectiveness in mass sport. To account for the nonlinear and time-varying characteristics of training effectiveness, this study proposed new mathematical assumptions and optimized parameters against individual longitudinal data. The external load (speed and wattage) and internal load (wearable-compatible heart rate variability [HRV] and heart rate recovery [HRR] related indicators) of each training day were collected for 28-42 days per person (420 paired data from 13 subjects during 12 weeks of medium-intensity continuous cycling). The longitudinal data were used to perform parameter estimation and model evaluation for each individual separately. When the optimal model output indicator was selected, the R^2^ values of the optimized model ranged from 0.61-0.95, with fitting root mean square error (RMSE) at 0.07-0.37, and mean absolute percentage error (MAPE) in predictive ability assessment at 3.99%-31.99%. However, a few individuals had larger fitting errors (minimum R^2^ of 0.32, maximum RMSE of 0.90) and predictive errors (maximum MAPE of 86.57%) when the output indicator was inappropriate. The original model generally has lower R^2^ and higher RMSE and MAPE. This shows the optimization of functional relationships and the application of individual longitudinal data have resulted in better performance of the model, but optimal indicator selection varies by individual. Furthermore, HRV and HRR related indicators are generalizable model output indicators that can be used to predict training effectiveness in mass sports through wearable devices and machine learning technology. However, the study has limitations including the homogeneous sample and single training type. Future research should validate the model across different sports and populations, investigating the factors affecting model fitting and prediction.”

It should be noted that, during the revision process, we identified a descriptive statistic error where the header row was mistakenly included as a training day in our initial count. This resulted in an overcount of the actual training days by one day for each subject. Consequently, the total session count in the abstract has been updated from 433 to 420 (details in the Response to Comment 7). Importantly, this correction does not impact the model outcomes, as all analyses were conducted using the raw session data.

Thank you again for your insightful feedback.

**Introduction**

Comment 2

The introduction section comprehensively covers the importance of training load monitoring and estimation models, clearly presents the historical development of FFM and gaps in the current literature. A literature summary on the Fitness-Fatigue Model and training effect discussions in mass sports is given in detail, and the topicality of the subject and the necessity of the study are clearly presented. The authors present a strong argument for the transition from the use of FFM in competitive sports to mass sports applications. Especially the argument on page 3, "The mathematical relationship of FFM requires optimization", establishes the justification of the study on a solid ground. The authors convey the basic problems in the field and the gaps in the literature in a logical order, and also emphasize the difference between the current approaches and their own suggestions.

The theoretical framework can be explained in more detail in the introduction section. In particular, the concepts of "adaptation" and "fatigue" should be addressed more comprehensively in the physiological context, and references to studies in different disciplines should be added. Additionally, a brief comparison of data-based model usage in mass sports with examples from different demographics or disciplines can be added to the introduction. The research hypotheses on page 5 can be reformulated in a specific and testable way.

Response:

Thank you for your positive feedback and valuable suggestions for enhancing our manuscript. We have carefully addressed each of your comments and made the following revisions to strengthen the introduction section:

We have added a dedicated passage to comprehensively explain the physiological concepts of "adaptation" and "fatigue" within the theoretical framework. Specifically, we inserted the following text into the second paragraph of the introduction:

“Physiologically, "adaptation" represents the positive functional and structural changes induced by training stimuli, such as an increase in muscle protein synthesis [12], mitochondrial-associated indices [13], and neuromuscular transmission efficiency [14]. Conversely, "fatigue" represents a temporary decline in bodily function that occurs after exercise, which may be related to factors such as muscle glycogen depletion [15], accumulation of metabolites [16, 17], and nervous system fatigue [18].”

The relevant references [12–18] have been included to support these explanations:

“12. Witard OC, Bannock L, Tipton KD. Making Sense of Muscle Protein Synthesis: A Focus on Muscle Growth During Resistance Training. International journal of sport nutrition and exercise metabolism. 2022;32(1):49-61. doi: 10.1123/ijsnem.2021-0139.

13.	Hadjispyrou S, Dinas PC, Delitheos SM, Koumprentziotis IA, Chryssanthopoulos C, Philippou A. The Effect of High-Intensity Interval Training on Mitochondrial-Associated Indices in Overweight and Obese Adults: A Systematic Review and Meta-Analysis. Front Biosci. 2023;28(11):281. doi: 10.31083/j.fbl2811281.

14.	Tumkur Anil Kumar N, Oliver JL, Lloyd RS, Pedley JS, Radnor JM. The Influence of Growth, Maturation and Resistance Training on Muscle-Tendon and Neuromuscular Adaptations: A Narrative Review. Sports. 2021;9(5):59. doi: 10.3390/sports9050059.

15.	Vigh-Larsen JF, Ørtenblad N, Spriet LL, Overgaard K, Mohr M. Muscle Glycogen Metabolism and High-Intensity Exercise Performance: A Narrative Review. Sports medicine (Auckland, NZ). 2021;51(9):1855-74. doi: 10.1007/s40279-021-01475-0.

16.	Allen DG, Lamb GD, Westerblad H. Skeletal muscle fatigue: cellular mechanisms. Physiol Rev. 2008;88(1):287-332. doi: 10.1152/physrev.00015.2007.

17.	Fiorenza M, Hostrup M, Gunnarsson TP, Shirai Y, Schena F, Iaia FM, et al. Neuromuscular Fatigue and Metabolism during High-Intensity Intermittent Exercise. Medicine and science in sports and exercise. 2019;51(8):1642-52. doi: 10.1249/mss.0000000000001959.

18.	Becker KM, Goethel M, Parolini F, de Paula Silva V, Vilas-Boas JP, Ervilha UF. The Price of Force: Motor Unit Adaptation Mechanisms in Pain Versus Fatigue. Physiotherapy research international : the journal for researchers and clinicians in physical therapy. 2025;30(3):e70081. doi: 10.1002/pri.70081.”

To address the suggestion for data-based model usage in mass sports, we have added a transitional sentence in the third paragraph of the introduction:

“Therefore, the use of data-based models to optimize the effects of exercise interventions has gained attention [31-33].”

We have referenced multiple studies that highlight the application of data-driven models in different contexts. However, to avoid excessive elaboration that might detract from the clarity of our research focus, we have not detailed these studies. The new references added are as follows:

“31. Mistiri ME, Rivera DE, Klasnja P, Park J, Hekler E. Model Predictive Control Strategies for Optimized mHealth Interventions for Physical Activity. Proc Am Control Conf. 2022;2022:1392-7. doi: 10.23919/acc53348.2022.9867350.

32.	Vandelanotte C, Trost S, Hodgetts D, Imam T, Rashid MDM, To QG, et al. MoveMentor-examining the effectiveness of a machine learning and app-based digital assistant to increase physical activity in adults: protocol for a randomised controlled trial. Trials. 2025;26(1):233. doi: 10.1186/s13063-025-08926-3.

33.	Kozan Cikirikci EH, Esin MN. The impact of machine learning on physical activity-related health outcomes: A systematic review and meta-analysis. Int Nurs Rev. 2025;72(2):e70019. doi: 10.1111/inr.70019.”

Regarding the research hypotheses, we have refined them to enhance specificity and testability. The revised hypotheses are now presented in a more structured format, with each hypothesis clearly defined to ensure they are concrete and measurable. The revised hypotheses are as follows:

“We hypothesize that (1) the mathematical modeling in this study will optimize the relationship between adaptation and fatigue, improving model interpretability through physiologically meaningful parameters; (2) HRV and HRR will serve as applicable and generalizable model output indicators for mass sports; (3) The model will demonstrate superior performance to the original model fitting with the higher fitting effect and lower prediction error through longitudinal data. It will also be capable of offering personalized evaluation and prediction of training outcomes, thereby offering effective guidance for improving sports performance and physical fitness in the general population.”

We sincerely appreciate your insightful feedback and hope that these revisions adequately address your suggestions regarding the introduction section and contribute to improving the overall quality of our manuscript.

**Methods**

Comment 3

The research design of the study has been meticulously conveyed; the selection of the sample, the training protocol and the data collection processes have been presented clearly and transparently. The use of scientifically accepted objective measurement techniques (HRV, HRR, speed, watts etc.) in both internal and external load measurements is quite strong in terms of methodology. In addition, the fact that the ethics committee approval and informed consent have been obtained is appropriate in terms of academic ethics. The statistical methods and model validation criteria (R^2^, RMSE, MAPE etc.) used in data analysis have been well selected and explained. The authors have clearly stated the model assumptions and explained the mathematical equations step by step. The systematic presentation of the equations (1)-(14) on pages 5-7 facilitates understanding the mathematical basis of the model. In particular, the addition of specificity coefficients (a and f) to model individual differences is one of the original contributions of the model.

In addition, the method of determining the individual parameters in the modeling process and the MATLAB codes should be explained in more detail. Data preprocessing steps and parameter optimization processes should be clearly stated so that the reader can repeat the study. The physiological meanings of each parameter (a, τ_a_, K_a_, C_1_, f, τ_f_, K_f_, C_2_) in equations (7) and (8) should be explained in more detail. The value ranges of these parameters and the details of the optimization algorithm used in their determination (other than least squares) should be given. In addition, alternative approaches used to assess the validity of the mathematical model should be mentioned.

Response:

Thank you for your constructive feedback and the insightful suggestions that will help us enhance our manuscript. We have carefully addressed each of your comments and made the following revisions:

In response to your suggestion, we have provided a more detailed account of the parameter optimization process in Section 2.3.1, including specifics on the optimization algorithm and its configuration. We believe these additions now provide sufficient detail to support reproducibility by readers. The revised text reads:

“Using the first 80% of training day data from each subject as the model learning database, model fitting and parameter estimation were performed using MATLAB 2021a (MathWorks, USA). Three internal load indicators (ΔHRR1、HRr%、TL_HRV_) were used to calculate the output indicators of the model for fitting separately, as detailed in Section 2.2.3. These output indicators were then used in the cost function. The parameters were estimated by minimizing the sum of squared errors (SSE) between model-predicted values and observed values, using nonlinear least-squares minimization via the Trust-Region algorithm within the Curve Fitting Toolbox. The optimization process utilized the toolbox's default configuration, including automatic initial parameter generation and standard convergence criteria. Initial conditions were handled by treating the integration constants *C_1_* and *C_2_* as adjustable parameters, which were estimated together with the other model coefficients (*a*, *τ_a_*, *K_a_*, *f*, *τ_f_*, *K_f_*). The fitted model surfaces were visualized as three-dimensional plots. The goodness of fit was evaluated using the coefficient of determination (R^2^), SSE, and root mean square error (RMSE).”

To address the need for a clearer explanation of each parameter's physiological meaning, we have added a new table (Supplementary Table S1) that provides a physiological interpretation of each parameter in Equations (10). The table is introduced in the text with the sentence: "The physiological interpretation of each parameter in Equations (10) is provided in S1 Table." The specific content of this table is:

**S1 Table. Physiological interpretation of optimized FFM parameters**

| Parameter | Symbol | Physiological Interpretation |
| --- | --- | --- |
| Adaptation decay rate | *a* | A larger value indicates faster adaptation loss and accounts for individual differences in adaptation |
| Adaptation time constant | *τ_a_* | A larger value indicates slower adaptation changes |
| Adaptation gain coefficient | *K_a_* | A larger value indicates greater adaptation per unit of external load |
| Adaptation initial constant | *C_1_* | Represents the baseline adaptation level before training begins |
| Fatigue decay rate | *f* | A larger value indicates faster fatigue recovery and accounts for individual differences in fatigue |
| Fatigue time constant | *τ_f_* | A larger value indicates slower fatigue changes |
| Fatigue gain coefficient | *K_f_* | A larger value indicates greater fatigue per unit of external load |
| Fatigue initial constant | *C_2_* | Represents the baseline fatigue level before training begins |

Regarding the requests for more information on data preprocessing and alternative model validation methods, we have made additional revisions that will be detailed in our response to Comment 5.

We sincerely appreciate your guidance and are confident that these revisions have significantly improved the clarity, depth, and scientific rigor of our manuscript.

Comment 4

The study protocol, inclusion/exclusion criteria, and ethical approval details are clearly stated. The methodological design of the study (12 weeks, 3 times a week, 30 minutes per session) complies with scientific standards. The measurement and calculation methods of internal and external load indicators (RMSSD, ΔHRR1, HRr%) are explained in detail on pages 8-9.

The validity and reliability information of the IPAQ questionnaire used to determine the physical activity levels of the participants should be added. The technical specifications, measurement precision, and validity and reliability details of the POLAR H10 heart rate band and ergoselet100 power bike used in the data collection process should be provided. The testing process in Figure 1 should be supported by a more detailed timeline. The formula or method used to determine the training intensity (HRmax 60-85%) on page 8 should be explained.

Response:

Thank you for your helpful suggestions. We have addressed each comment and made the following revisions:

We have added a brief description of the International Physical Activity Questionnaire's validity and reliability in the text, and the relevant references [39-41] have also been added. The updated version is as follows:

“Subjects’ characteristics such as age, gender, BMI, and pre-experiment physical activity level (using the International Physical Activity Questionnaire long roll, a reliable and valid tool for physical activity assessment [39-41]) were collected before formal training.”

The new references added are as follows:

“39. Craig CL, Marshall Al Fau - Sjöström M, Sjöström M Fau - Bauman AE, Bauman Ae Fau - Booth ML, Booth Ml Fau - Ainsworth BE, Ainsworth Be Fau - Pratt M, et al. International physical activity questionnaire: 12-country reliability and validity. Medicine and science in sports and exercise. 2003;35(8):1381-95.

40.	Hagströmer M, Oja P Fau - Sjöström M, Sjöström M. The International Physical Activity Questionnaire (IPAQ): a study of concurrent and construct validity. Public Health Nutr. 2006;9(6):755-62.

41.	Kim Y, Park I Fau - Kang M, Kang M. Convergent validity of the international physical activity questionnaire (IPAQ): meta-analysis. Public Health Nutr. 2013;16(3):440-52.”

To address the request for technical details, we have added concise descriptions and provided references to the official product pages and supporting literature:

“In this study, the model input indicator is the external load collected during the each training session using an ergoselect100 power bicycle, a classic ergometer for exercise tests [43].”

“POLAR H10 heart rate band, a reliable and valid device for heart rate monitoring [56, 57], was used to obtain the root mean square of the successive differences (RMSSD) of the difference between adjacent R-R intervals.”

The new references added are as follows:

“43.	GmbH E. ergoselect 100 – Bicycle Ergometer [Product technical page] [Product specifications]. Ergoline GmbH; 2025 [Cited 2025 July 14]. Available from: <https://www.ergoline.com/en/__productdetails/artn-ergoselect-100.html>.

56.	Oy PE. H10 Heart Rate Sensor - Technical Specifications [Product page] [Product specifications]. Polar Electro Oy; 2025 [cited 2025 July 16]. Available from: https://www.polar.com/en/sensors/h10-heart-rate-sensor.

57.	Schaffarczyk M, Rogers B, Reer R, Gronwald T. Validity of the Polar H10 Sensor for Heart Rate Variability Analysis during Resting State and Incremental Exercise in Recreational Men and Women. Sensors. 2022;22(17):6536. doi: 10.3390/s22176536.”

What’s more, we have redesigned Figure 1 to include a detailed timeline, which now visually outlines the testing process. Additionally, to address feedback from another reviewer regarding the structuring of the data acquisition process, we have comprehensively revised the section to align with the schematic order presented in Figure 1. This involved transitioning the structure from a classification based on indicators to one based on testing phases, thereby improving the logical flow and readability of the data collection methods. These specific revisions can be found in the manuscript under section “2.2.3 Data Collection Methods.”

Finally, we have clarified the method for determining training intensity by adding:

“Prior to formal training, each participant underwent an incremental load cycling test to determine their individual HR_max_. Based on this, the target heart rate range for training was set at 60–85% of each participant's HR_max_ [42].”

We appreciate your helpful feedback and are confident that these revisions have significantly improved the clarity and completeness of our manuscript.

Comment 5

The authors have clearly stated the statistical approaches used for parameter estimation, model fit, and prediction ability assessment. The separation of model learning (80%) and test (20%) databases is methodologically appropriate. Various metrics (R^2^, SSE, RMSE, MAPE) were used to evaluate model performance. The rationale and potential effects of the choice of data normalization (in the range of 0.1-1) should be discussed. More technical details about the specific implementation of the least squares method and the optimization process should be provided. Statistical tests used to compare models (significance tests, Bayesian approaches, etc.) should be specified. The reliability of the results can be increased by performing robustness analysis of the parameters with bootstrap or cross-validation.

Response:

Thank you for your positive feedback and suggestions. We have revised the statistical analysis section of the manuscript based on your feedback.

In response to your query regarding the data normalization range of 0.1-1, we would like to clarify that this specific range was selected to prevent potential division by zero errors in the model output indicator calculation. Given that the model output indicator is the ratio of external load to internal load, a value of zero in the internal load component (denominator) would result in an undefined output. By normalizing the data to the 0.1-1 range, we ensure mathematical validity while preserving the relative scale differences between data points. The transformation maintained the relative scale differences, ensuring that the proportional relationships between data points were preserved. This approach not only prevented division by zero but also enhanced the stability of the model. We have also revised the text of this section to make our purpose clearer. The revised version can be found in section 2.3 and is as follows:

“Each indicator (external load and internal load) is normalized with an interval of 0.1-1 using MATLAB 2021a (MathWorks, USA). This specific normalization range prevents potential division by zero errors in the model output indicator calculation. The transformation maintained relative scale differences while ensuring mathematical validity across all observations.”

Regarding the need for more technical details about the specific implementation of the least squares method and the optimization process, we have thoroughly enhanced Section 2.3.1. The modified Section 2.3.1 reads as follows:

“Using the first 80% of training day data from each subject as the model learning database, model fitting and parameter estimation were performed using MATLAB 2021a (MathWorks, USA). Three internal load indicators (ΔHRR1、HRr%、TLHRV) were used to calculate the output indicators of the model for fitting separately, as detailed in Section 2.2.3. These output indicators were then used in the cost function. The parameters were estimated by minimizing the sum of squared errors (SSE) between model-predicted values and observed values, using nonlinear least-squares minimization via the Trust-Region algorithm within the Curve Fitting Toolbox. The optimization process utilized the toolbox's default configuration, including automatic initial parameter generation and standard convergence criteria. Initial conditions were handled by treating the integration constants *C_1_* and *C_2_* as adjustable parameters, which were estimated together with the other model coefficients (*a*, *τ_a_*, *K_a_*, *f*, *τ_f_*, *K_f_*). The fitted model surfaces were visualized as three-dimensional plots. The goodness of fit was evaluated using the coefficient of determination (R^2^), SSE, and root mean square error (RMSE).”

Regarding your comment on “specifying the statistical tests used for model comparison”, we have added paired tests to compare the performance of the optimized and original models. The specific methods are detailed in Section 2.3.2 of the Methods:

“To further evaluate the performance of the fitted model, the above indicators were compared with the same indicators’ values obtained from the FFM original model before optimization [20, 38]. The normality of differences was assessed using the Shapiro-Wilk test. Paired t-tests (for normally distributed differences) or Wilcoxon tests (for non-normally distributed differences) were then performed, with Bonferroni correction for multiple comparisons. All analyses were conducted using MATLAB 2021a (MathWorks, USA) at a significance level of p<0.05.”

We have also modified Figures 2 and 3 to include markers of statistical significance. Furthermore, we describe the results of these statistical tests in Section 3.2 of the Results:

“The fitting effect of the original model is significantly worse than that of the optimized fitting model in this study. Statistical analysis revealed significant increases in R^2^ for ΔHRR1 (Bonferroni-corrected *p*=0.030) and HRr% (Bonferroni-corrected *p*=0.021). Moreover, most fitting indicators showed consistent positive trends in the optimized model, as presented in Fig 2.”

For the parameter robustness analysis, please refer to the response to Comment 9 for details, as it overlaps with that section.

Thank you again for your comments.

**Results**

Comment 6

The findings are presented clearly and supported by tables and graphs. The advantages of the model optimization over the original model are statistically demonstrated and reasonably conveyed with detailed results. The possible contribution of the study to individualized training processes is well-founded with the various parameter values obtained.

Further analysis and visual improvement of some tables and graphs in the text would increase the completeness of the report. In particular, the missing units, axis names and statistical significance indicators in the graphs should be clarified. In addition, In the findings section, possible limitations of the model (e.g. inter-individual variation, small sample bias) should be discussed more clearly.

Response:

Thank you for your positive and constructive feedback. We have revised tables and graphs in the text based on your suggestions and further refined the discussion on the model's limitations in the last paragraph. We have also rearranged and redrawn Figures 2 and 3, and added labels to indicate statistical significance, enhancing clarity and readability. **As this comment provides general advice on the results section, the specific revisions are detailed in our responses to comments 7-9 and 12.**

Additionally, since you specifically mentioned the missing units and axis name in the graphs in this comment, we would like to provide a brief clarification here to prevent misunderstandings. In this revision, we have checked the axis names and units, adding necessary units (e.g., for the T - axis in Figure 4). However, some axes (e.g., the W - and P - axes in Figure 4) lack units because they represent normalized values of external load levels or performance levels. Therefore, we have retained the original style for these axes.

Thank you again for your feedback. Please refer to the following text for detailed modifications to the results section.

Comment 7

Table 1 clearly presents the demographic and anthropometric characteristics (age, gender, BMI, physical activity level) and training completion status of the participants. A total of 433 paired training data from 13 participants provided sufficient data for model training. Additional sports risk monitoring measures for at-risk participants (overweight, low physical activity level) are methodologically sound. A more detailed summary statistics of participants’ mean age and standard deviation, gender distribution, BMI ranges, and physical activity levels can be provided. The statement “subjects’’ compliance was good” on page 11 should be supported by quantitative data (e.g., ratio of planned vs. actual training sessions). Although no dropouts are noted, possible reasons for missing data (illness, work/school conflicts, etc.) should be explained. The potential impact of differences in training duration between participants on model results should be discussed.

Response:

Thank you for your insightful feedback on the presentation of our participant data and study compliance. We have carefully addressed your comments and made the following revisions to enhance the clarity of our manuscript:

We have enhanced Table 1 by adding a "Summary Statistics" row at the bottom, which now includes the participants' mean age and standard deviation, gender distribution, and BMI ranges. Additionally, we have inserted an "Attendance Rate" column to the right of the table to provide quantitative data on subjects' compliance. In the text section 3.1, we have expanded the discussion of compliance and provided an explanation for possible reasons for absences

Moreover, during the revision of Table 1, we identified a minor error where the header row was mistakenly included as a training day in our initial count. This resulted in an overcount of the actual training days by one day for each subject. We have now corrected this error in Table 1 and the corresponding descriptive text (section 3.1 and abstract). Importantly, this correction does not impact the model outcomes, as all analyses were conducted using the raw session data. Additionally, in accordance with the journal's policy, we have provided all relevant subject training data in the supporting documents and referenced this data within the text of this section.

The revised section 3.1 is now read:

“Training data comprised paired external load and internal load measurements from each session, with 28–42 days of records per participant and a total of 420 paired data from 13 subjects. Due to the separate training of participants and the use of longitudinal data from each participant for personalized parameter estimation and model testing, the training completion status was different (Table 1). The Training Attendance Rate, defined as the ratio of actual training sessions attended to planned sessions and averaging 88.5±5.9% across all subjects, indicates excellent compliance among the participants. Most deviations from 100% attendance were attributed to menstrual cycles and the overlap of the final training week with the school winter break. Three overweight subjects (BMI ≥ 24kg/m^2^) and two subjects with low physical activity levels were given additional sports risk monitoring to prevent injury. None of the subjects withdrew from the study, further underscoring the good compliance throughout the research period. The detailed training data for each training session of all participants can be found in S1 File.”

Regarding the potential impact of differences in training period between participants on model results, we have incorporated a detailed discussion in the revised Discussion section. Although the overall training attendance rate in our study was high and the missing training sessions did not show a regular impact on the model fitting effect, we recognize that the variation in training period should not be overlooked.

We have included the following text at the end of the fifth paragraph of the discussion section:

“Additionally, the differences in training period between participants may have influenced the model performance. Although the overall training attendance rate in our study was high and the missing training sessions did not show a regular impact on the model fitting effect, the variation in training period should not be overlooked. Longer training periods could provide more data points for parameter estimation, potentially enhancing model accuracy. However, due to individual differences in training responses, changes in training period do not directly correspond to proportional changes in model performance. Future studies could investigate this relationship further by controlling for training period or including it as a covariate in the analysis. To better capture the comprehensive impact of training period differences on model performance, it is also recommended that future research employ larger sample sizes and collect data over longer timeframes.”

We believe that this addition addresses your concern and provides a more complete discussion of the factors that may influence the model results.

Comment 8

Figure 2 comprehensively visualizes the model fit metrics obtained using three different output indicators (ΔHRR1, HRr%, TLHRV). It is clearly shown that the optimized model provides a better fit than the original model. The majority of participants achieved moderate-high R² values (0.5-0.7 and >0.7) and low RMSE values.

The cut-off points used in interpreting the fit results on page 12 (0.5, 0.7 for R², etc.) should be justified based on literature. A more detailed analysis of individual differences should be provided - which participants showed better/worse fit on which indicators and possible reasons for this should be discussed.

Response:

Thank you for your valuable suggestions regarding the justification of the cut-off points used in interpreting the fit results and the analysis of individual differences.

After conducting a thorough literature review on the cut-off points for R^2^, we found that most existing studies report specific R^2^ values without explicitly defining universal cut-off points. Since there is no definitive consensus in the literature regarding these thresholds, we decided to revise the text to focus on the statistical distribution of the R^2^ values obtained in our study, rather than attempting to reference undefined thresholds. The revised paragraph now emphasizes the actual R² ranges observed across subjects, providing a clearer and more data-driven description of the model fitting results. This approach maintains scientific rigor while accurately communicating the outcomes of our research.

The specific text we have added is as follows:

“When utilizing ΔHRR1 as a factor in calculating the output indicators of the model, the evaluation of the model fitting effect showed that all 13 subjects obtained small SSE and RMSE, with R^2^ values ranging from 0.54 to 0.84 (54% of subjects exhibited R^2^ values between 0.50 and 0.70, and 46% of subjects exhibited R^2^ values between 0.70 and 0.90). As for HRr%, the majority of subjects achieved good model fitting effect and low error, but there were also four subjects with R^2^ values less than 0.50. When it comes to TL_HRV_, most subjects achieved good fitting results, with R^2^ values ranging from 0.40 to 0.95 and RMSE being low. However, three individuals had slightly high SSE. The fitting effect of the original model is significantly worse than that of the optimized fitting model in this study. Statistical analysis revealed significant increases in R^2^ for ΔHRR1 (Bonferroni-corrected p=0.030) and HRr% (Bonferroni-corrected p=0.021). Moreover, most fitting indicators showed consistent positive trends in the optimized model, as presented in Fig 2. The SSE, RMSE, and R^2^ values obtained through fitting with different output indicators are also shown in Fig 2. Specific values for each subject are provided in S5-S7 Tables.”

Regarding the analysis of individual differences, we have also dedicated significant attention to this aspect. In the fourth and fifth paragraphs of the discussion, we have extensively explored the possible reasons for individual differences in model fitting by integrating the model fitting results, participant characteristics, and relevant existing studies. However, we acknowledge that we have not yet identified consistent factors contributing to these individual differences, which makes it difficult to present the analysis of individual differences as statistical results in the result section. We have recognized this limitation in the final paragraph of the discussion, where we have also highlighted the need for future research to address this gap.

We believe that identifying optimal output indicators for individuals and further investigating the factors affecting model fitting and prediction are crucial directions for future research. As such, we have explicitly suggested in the final paragraph of the discussion that:

“Future research should focus on identifying optimal output indicators for individuals. This would provide a more robust foundation for selecting model indicators and achieving more stable fitting results. Additionally, the factors affecting model fitting and prediction need to be studied further. Future studies should collect more confounding factors to explore and eliminate those that may introduce "noise" into the analysis. This will serve as a basis for further refining the model and enhancing its practical application.”

We hope these revisions adequately address your concerns and strengthen the manuscript.

Comment 9

Figure 3 clearly shows the model's predictive ability with MAPE and RMSE metrics. The optimized model has been shown to have better overall prediction performance. Figure 4 effectively visualizes the prediction performance of the model for Participant 4 by presenting 3D model images from different visual angles. While the chronological nature of the test database (last 20% training day data) used in the prediction capability assessment is appropriate for testing the model’s true prediction capacity over time, it is recommended that cross-validation approaches (k-fold or leave-one-out) be used to increase the robustness of the results. The change in prediction error over time should also be examined – it is important to see how well the model can predict the distant future compared to the near future. The statement on page 12 that “Although the prediction error of some subjects' fitting models is slightly higher than that of the original model, …” requires a specific analysis of these cases.

Response:

Thank you for your insightful comments and suggestions. We have carefully considered each of your points and made appropriate revisions to the manuscript. Below is a detailed account of the changes we have implemented.

Regarding the suggestion to use cross-validation approaches to increase the robustness of the results, we have carefully deliberated and decided to retain the original method of using the last 20% of training day data for prediction capability assessment. Our reasons are as follows:

(1) The sequential 80% and 20% split we employed strictly maintains temporal causality, which is crucial for our model that incorporates temporal parameters. This approach aligns with the real-world application of sports training monitoring where only past data is available to predict future outcomes. Traditional cross-validation methods, which involve random data splitting, would violate the temporal order and thus are not suitable for our model.

(2) Our study utilizes individual longitudinal training datasets for personalized validation and model analysis. This means that the model was not merely validated on a single dataset but across 13 individuals with three different model output indicators, resulting in validation across 39 datasets. This extensive validation provides substantial evidence of the model's robustness.

However, we fully agree with your suggestion to examine the change in prediction error over time. Therefore, we have added a temporal dependency analysis to assess how well the model can predict the distant future compared to the near future. The methodology for this analysis is detailed in Section 2.3.2:

“Moreover, temporal dependency analysis using Spearman rank correlation analysis was used to investigate the relationship between prediction horizon (days since last training day in the model learning database) and absolute percentage error, assessing whether prediction errors systematically vary with temporal distance. Statistical significance was determined at p<0.05.”

In the results section (Section 3.3) and Supporting Tables, we have presented the findings from the temporal dependency analysis. Furthermore, based on these results, we have included a discussion on the temporal dependency analysis in the third paragraph of the discussion section:

“Moreover, temporal dependency analysis in this study further revealed that the temporal dependency of prediction errors lacks population-wide consistency, with only a few individuals showing significant but directionally inconsistent correlations. This suggests that the sensitivity of prediction accuracy to temporal distance is highly individualized. Future research could explore adding a "time-adaptive module" to exercise monitoring platforms. This module can determine whether an individual's model predictions exhibit temporal dependence based on their training data. For individuals with negative correlations, longer training cycles could be adopted, while those with positive correlations might benefit from high-frequency short cycles. This is crucial for enhancing the scientific nature of training in mass sports.”

In response to the comment on individual subject variability, please refer to our detailed response to Comment 8 for further clarification, as there is overlap between the two.

Thank you again for your valuable feedback.

**Discussion**

Comment 10

The discussion section is logically structured around the optimization of the model, the use of longitudinal data, and the general applicability of the model output indicators. The potential use of the proposed model in the field, the future place of personalized approaches in sports with technology are successfully addressed. In addition, the contribution of the model to the training processes in mass sports is compared with existing methods and its importance is ranked. The authors comprehensively relate their findings to the existing literature and effectively discuss unique concepts such as "HRV fingerprints". The discussion on the causes of individual differences on pages 16-17 is particularly in-depth.

The discussion can address the practical applications and potential impact of the model from a broader perspective. In particular, more detailed suggestions should be provided for the dissemination of the model through wearable technologies and smartphone applications. It would also be useful to evaluate the effects of sample diversity and demographic variables such as age/gender on the findings in more detail in the discussion. A brief assessment of the applicability of the model in clinical or professional sports environments can also be added. The section on the limitations of the study on page 18 should be expanded, and the effects of factors such as sample size, demographic diversity, and type of training (only cycling) on the generalizability of the results should be discussed. In addition, precautions against the risk of overfitting should be explained in more detail; future multicenter, randomized studies should be recommended to validate the model. It should be clearly emphasized that the model should be retested with different branches and age groups. Concrete recommendations for future research (model validation on different sports, age groups, genders, theoretical mechanisms) should be presented in more detail.

Response:

We sincerely appreciate your positive feedback on the discussion section and your constructive suggestions for further improvement. We have meticulously addressed the following aspects in our revision:

Firstly, regarding the broader perspective on the model's practical applications and potential impact, we fully agree with your viewpoint. Indeed, the extended application of the model, particularly its integration with wearable devices and smartphone applications, has been an ongoing focus of our research. In response to your suggestion, we have elaborated on the model's practical applications prospects and its potential influence in the final part of the third paragraph of the discussion. We have also briefly assessed the model's applicability in clinical and professional sports settings, while keeping the focus on mass sports as the study's primary emphasis.

The specific text we have added is as follows:

“Additionally, the integration of machine learning empowers the model with the capability to identify an individual's "fingerprint" based on their training data, thereby facilitating personalized evaluation. Founded on real-time training data acquisition through wearable devices, the model incorporates machine learning to continuously learn and capture individual physiological characteristics. This approach establishes a foundation for developing adaptive training monitoring software [61]. Once integrated into smartphone applications, such software can provide users with an intuitive interface, offering personalized training adjustment recommendations, such as intensity modulation and rest advice. Moreover, temporal dependency analysis in this study further revealed that the temporal dependency of prediction errors lacks population-wide consistency, with only a few individuals showing significant but directionally inconsistent correlations. This suggests that the sensitivity of prediction accuracy to temporal distance is highly individualized. Future research could explore adding a "time-adaptive module" to exercise monitoring platforms. This module can determine whether an individual's model predictions exhibit temporal dependence based on their training data. For individuals with negative correlations, longer training cycles could be adopted, while those with positive correlations might benefit from high-frequency short cycles. This is crucial for enhancing the scientific nature of training in mass sports. Meanwhile, this application model also holds significant promise in both clinical and competitive sports fields. In clinical sports rehabilitation, customizing training loads according to individual recovery situations in a safer and more precise manner can maximize rehabilitation efficacy and minimize injury risks [62], which is consistent with the application outlook of optimizing the model in this study. As for competitive sports, given that the FFM originated in this domain, its combination with machine learning can further provide a basis for precise training.”

The references added are:

“61. Weyns D, Schmerl B, Kishida M, Leva A, Litoiu M, Ozay N, et al., editors. Towards Better Adaptive Systems by Combining MAPE, Control Theory, and Machine Learning. 2021 International Symposium on Software Engineering for Adaptive and Self-Managing Systems; 2021 18-24 May 2021.

62. Taylor JB, Ford KR, Queen RM, Owen EC, Gisselman AS. Incorporating Internal and External Training Load Measurements in Clinical Decision Making After ACL Reconstruction: A Clinical Commentary. Int J Sports Phys Ther. 2021;16(2):565-78. doi: 10.26603/001c.21152.”

Regarding the limitations section and suggestions for future research, we have made the following revisions: We expanded the discussion to address the limitations of the study, including the effects of sample size, demographic diversity, and the exclusive focus on cycling as the training type. We also provided a more detailed explanation of the precautions taken against the risk of overfitting and recommended future multicenter, randomized studies to validate the model. Furthermore, we emphasized the need to retest the model with different branches and age groups and presented more concrete recommendations for future research.

The revised paragraph in the discussion section is as follows:

“Overall, this study has several limitations that should be acknowledged. While the model demonstrated good fitting and prediction effects, it lacks an in-depth exploration of the personalized differences observed in various output indicators. Future research should focus on identifying optimal output indicators for individuals. This would provide a more robust foundation for selecting model indicators and achieving more stable fitting results. Additionally, the factors affecting model fitting and prediction need to be studied further. Future studies could collect more confounding factors to explore and eliminate those that may introduce "noise" into the analysis. This will serve as a basis for further refining the model and enhancing its practical application. Moreover, the sample used in this study is homogeneous (average age 22±3 years) and only involves cycling as the training type. This limits the generalizability of the findings to other age groups and training types. Future research should validate the model across different sports, age groups, and genders to ensure its reliability and effectiveness in diverse populations. To address the risk of overfitting, future studies should also employ rigorous cross-validation techniques and consider incorporating additional variables that may influence model outcomes. Multicenter and randomized studies are also recommended to further establish the model’s validity and applicability across different contexts. Finally, the current model does not account for overtraining. Future research could investigate extreme autonomic responses, such as paradoxical changes in HRV, to enhance the model’s ability to detect and interpret specific training states.”

We believe these revisions have strengthened the manuscript and provided a clearer direction for future research. We appreciate your guidance in improving the quality of our work.

**Conclusion**

Comment 11

The conclusion section concisely summarizes the main findings of the study and highlights the potential of the model to predict training effectiveness in mass sports. It is clearly stated that the optimized model performs well thanks to the use of functional relationships and individual longitudinal data.

The conclusion section should emphasize the theoretical and practical contributions of the study more strongly. Concrete steps and recommendations for future applications of the model should be added. The phrase “combined with wearable devices and machine learning” should be expanded with more specific examples. The conclusion should position the significance of the study in a broader context for the scientific community, coaches, athletes, and the general population.

Response:

Thank you for your feedback on the conclusion section. We have carefully revised the conclusion to better align with your suggestions and enhance the presentation of our study's contributions.

We have specified that this study provides a foundation for applying the model in wearable technology. Additionally, we have expanded on the phrase “combined with wearable devices and machine learning” by illustrating how such software could offer potential applications for the general population, coaches, and rehabilitation physicians.

The revised conclusion now reads as follows:

“The good performance of the model is attributed to the optimization of functional relationships and the application of individual longitudinal data. HRV and HRR related indicators are generalizable model output indicators that can be used to predict training effectiveness in mass sports, though individual differences in optimal indicators selection exist. This study provides a foundation for applying the model in wearable technology, offering a basis for developing adaptive training monitoring software. Such software, when integrated into smartphones, can provide users with personalized training recommendations, offering potential applications for general population, coaches, and rehabilitation physicians.”

Thank you again for your insightful and meticulous feedback.

**Figures and tables**

Comment 12

Figure 1 clearly visualizes the testing process. Figures 2 and 3 effectively present model performance metrics. The 3D visualizations in Figure 4 illustrate the predictive capacity of the model from various perspectives. Table 1 provides a comprehensive overview of the participants’ characteristics. Additional tables (S1-S12) provide detailed information on model parameters, fit, and prediction results.

Figure 1 can be redesigned in a timeline format to show data collection points more clearly. Additional explanations and arrows can be added to the 3D visualizations in Figure 4 to provide a better understanding of model features. Summary statistics (mean, standard deviation, range) can be added to Table 1. Additional tables should provide more descriptive information and context.

Response:

Thank you for your suggestions regarding improvements to figures and tables. We have implemented the following changes:

Figure 1 has been redesigned in a timeline format to more clearly illustrate the data collection points throughout each training session.

As for Figure 4, based on your suggestion, we have carefully revised it. We realized that the original figure contained too many variable names, which might have made it difficult to understand. Therefore, we have simplified the figure by removing excessive variable names, revised the legend and caption to make them more concise and informative, and added explanatory arrows. We believe these revisions have significantly enhanced the clarity and interpretability of Figure 4. We appreciate your guidance in improving the quality of our work.

In Table 1, we have added a “Summary Statistics” row at the bottom, which includes the participants’ mean age and standard deviation, gender distribution, BMI ranges, and physical activity levels. Additionally, an “Attendance Rate” column has been inserted to the right of the table.

Regarding your suggestion that “Additional tables should provide more descriptive information and context”, we have added Supplementary Table S1 in the Additional tables to offer a clearer explanation of the physiological meaning of each parameter. For more details, please refer to our response to Comment 3.

We hope these revisions enhance the clarity of our figures and tables.

**References**

Comment 13

The bibliography is comprehensive and up-to-date, covering key literature in the areas of training load monitoring, FFM, HRV, and HRR. Citations are used appropriately in the text.

More resources could be cited on the integration of machine learning and FFM. More up-to-date references on wearable technologies could be added. Existing citations could include more meta-analyses and systematic reviews.

Response:

Thank you for your valuable suggestions regarding the bibliography. We have incorporated additional references to further strengthen the literature base of our manuscript throughout the revision process, including more meta-analyses and systematic reviews as you recommended. These additional citations have been detailed in our responses to the above specific comments.

Thank you again for your comprehensive feedback.

# To Reviewer 2:

General response

Thank you for your positive evaluation of our manuscript and the constructive suggestions provided. We sincerely appreciate the opportunity to enhance our work based on your expert feedback.

We have made comprehensive revisions to improve the clarity and quality of our manuscript. The methodology section has been refined, and the description of the model optimization process has been enhanced. The data collection and testing sections have been restructured to follow a schematic order. Reference formats have also been checked and corrected.

For detailed information on all modifications, please refer to our point-by-point responses below.

Thank you again for your insightful feedback.

Point-by-point responses

Comment 1

The manuscript by Wang et al. proposes a method to optimize the mathematical relationship between "adaptation" and "fatigue" of traditional fitness-fatigue model (FFM). The study may provide useful information by adapting the model to the subject characteristics but the methodology needs several clarifications, as well as manuscript structure needs improvement. Description of the model and the related optimization procedure need revisions to improve clarity. Description of signals and data acquired, as well as tests performed for the acquisition, should follow a schematic order. A list of major and minor comments is reported in the following.

Response:

Thank you for your thorough evaluation. We have carefully considered your comments regarding the need for clarification in the methodology and structural improvements to the manuscript. In response to your advice, we have made detailed enhancements to the methodological section, particularly in the description of the data acquisition and model optimization procedure.

Regarding the data collection and testing, we have thoroughly revised the structure as per your recommendation to follow a schematic order as illustrated in Figure 1. This revision involves shifting the classification from indicators to testing phases, which improves the clarity of our methodology. The detailed modifications can be found in section “2.2.3 Data Collection Methods” of the revised manuscript.

Regarding the revisions to the model description and optimization procedure, we have made improvements to enhance clarity. **For specific details on these and other related modifications, please refer to the responses below, as there is some overlap in content.**

Comment 2

- Ref. 6. Please check since it is not correctly formatted.

Response:

Thank you for your attention to detail. We have checked Reference 6 and found that it was missing the page numbers. We have now added the necessary information, and its format is updated as follows:

“6.	Carl F, A R-MJ, J dKJ. Monitoring Training Loads: The Past, the Present, and the Future. International journal of sports physiology and performance. 2017;12(Suppl 2):S22-S8. doi: 10.1123/ijspp.2016-0388.”

In addition, we have conducted a thorough review of all other references, adding any missing page numbers and ensuring consistency. We have also downloaded the “PLoS.ens” reference format from the PLOS official website and applied it using EndNote software to standardize the references.

It is worth noting that changes made to the references through EndNote do not seem to be captured by the track changes mode. Therefore, the reference section still appears in black font and does not highlight the modifications. We hope this will not affect your review process.

Thank you again for your careful reminder.

Comment 3

- Lines 14-16 The sentence is incomplete. Please rephrase.

Response:

Thank you for your comment regarding the sentence in the abstract. We have carefully reviewed the sentence and revised it for improved grammatical completeness. The updated version is as follows:

“The aim of this study was to optimize the mathematical relationship between "adaptation" and "fatigue" in the FFM, identify generalizable model output indicators, and evaluate its performance in predicting training effectiveness in mass sport.”

We appreciate your attention to detail and hope this revision addresses your concern.

Comment 4

- Lines 17-18 The terminology used in not appropriate and focused for mathematical modeling domain.

Response:

Thank you for pointing out this issue in the abstract. We agree that the phrase “using mathematical language” was too general and did not clearly describe our methodology. To address your concern, we have revised the relevant sentence in the abstract as follows:

"To account for the nonlinear and time-varying characteristics of training effectiveness, this study proposed new mathematical assumptions and optimized parameters against individual longitudinal data."

Additionally, we have updated the corresponding sentence in Section 2.1 of the main text to ensure consistency throughout the manuscript.

We appreciate your insightful feedback, which has helped us improve the clarity and accuracy of our description.

Comment 5

- Lines 70-71. This sentence is not clear to me. Please clarify.

Response:

Thank you for noting that the sentence was unclear. We have now revised the sentence in the third paragraph of the Introduction as follows:

“The mathematical structure of FFM allows its parameters to be estimated by machine learning algorithms [21]. With sufficient data accumulation, the model can provide personalized guidance based on individual characteristics, thereby enhancing sports performance and preventing sports injuries.”

This revision clearly links the model's mathematical structure to parameter estimation using machine learning, and then to the resulting personalized training guidance. We hope this clarifies the intended meaning.

Comment 6

- Lines 89-91. The concept of internal and external loads should be better described.

Response:

Thank you for your suggestion. In the revised manuscript, we have added a brief explanation in the fourth paragraph of the Introduction to clarify these concepts:

“Moreover, training load comprises both external and internal components [36]. External load refers to the predetermined physical stress imposed on an individual during training, such as exercise intensity and volume. Internal load, on the other hand, reflects the physiological responses of the individual to this external stress [37]. Accordingly, for model input and output indicators, we utilized the power bicycle to collect external load and selected heart rate variability (HRV) and heart rate recovery (HRR) related indicators that reflect autonomic nervous activity as internal load indicators.”

This addition provides a succinct and comprehensive overview of internal and external loads. We believe this revision addresses your concern.

Comment 7

- Section 2.1. It is important to clarify what has been taken from Banister et al. and what has been modified.

Response:

Thank you for your insightful comment on clarifying the distinctions between the original FFM and our optimization. We recognized that the initial wording did not clearly articulate what we retained from the original FFM and what we added. In response, we have thoroughly revised the relevant section in Section 2.1 to detail the relationship of each modeling assumption to the original FFM. We believe these revisions effectively address the previous shortcomings. The revised assumptions now explicitly state:

“Based on the nonlinear and time-varying characteristics of training effectiveness, this study proposed new mathematical assumptions and formulated them into an optimized mathematical model. The specific modeling assumption are as follows:

(1)	During training, fatigue (*F*) and adaptation (*A*) changes are primarily driven by external loads, and there is no occurrence of overtraining. This study retains the core concept of the FFM originally proposed by Banister et al., which describes performance as a function of adaptation and fatigue resulting from external loads [11, 20, 38]. However, to avoid the complexity associated with overtraining responses, this assumption is added to ensure the model focuses on the typical training response.

(2)	External loads will have a beneficial effect on adaptation and fatigue, while adaptation and fatigue will fade over time, and the rate of decline is related to the individual's adaptation and fatigue level. To address the limitations of the traditional FFM, this study incorporates nonlinear dynamics to optimize the mathematical relationship between adaptation and fatigue, differing from the first-order linear equations used in the original model [11, 21]. This approach better reflects the real-world training response, accounting for the nonlinear nature of exercise training and the time-dependent fading of adaptation and fatigue.

(3)	The change in performance level is reflected by the difference between adaptation and fatigue, a principle retained from the original FFM [11, 20, 38]. Additionally, this study introduces specificity coefficients to better capture individual differences in adaptation and fatigue dynamics, enabling the model to provide more personalized guidance based on individual characteristics.”

Thank you again for your valuable suggestion.

Comment 8

- Eq.(5) and eq. (6). Is this accounting for the initial conditions of the two differential equations?

Response:

Thank you for highlighting the need to clarify how the initial conditions are handled in Eq. (5) and Eq. (6). In the revised manuscript we have made the following changes:

Immediately after Eq. (6) we added:

“The integration constants *C_1_* and *C_2_* were determined by estimating them alongside the model coefficients, based on the initial conditions (see Section 2.3.1 for estimation details).”

In the parameter estimation section (Section 2.3.1) we appended: “Initial conditions were handled by treating the integration constants C_1_ and C_2_ as adjustable parameters, which were estimated together with the other model coefficients (*a*, *τ_a_*, *K_a_*, *f*, *τ_f_*, *K_f_*).”

These revisions explicitly link the integration constants to the initial conditions and describe how the latter were estimated, which we hope resolves your concern.

Comment 9

- Lines 155-156. 85% HR_max_ usually refers to vigorous and not moderate exercise.

Response:

We appreciate your guidance on this matter and have carefully considered the rationale behind our intensity range selection.

The target heart rate range of 60–85% HR_max_ for moderate-intensity cycling in this study was chosen based on two key considerations. First, it aligns with the recommendations of the National Fitness Guide of the Chinese General Administration of Sport, and the relevant reference has been inserted into the manuscript. This guide, designed for the general public's daily fitness activities, specifically designates the moderate-intensity heart rate zone as 60–85% HR_max_. Given our study’s focus on mass sports, adopting this range ensures practical relevance and applicability to our target population.

Second, we acknowledge that our initial manuscript did not adequately describe the individualized HR_max_ testing procedure we used, which might have caused confusion. The HR_max_ values used to define this range were not estimated using predictive formulas like the commonly cited "220 minus age" equation. Instead, they were directly determined from each participant's individual incremental load cycling test conducted prior to the training program. This approach accounts for individual differences in cardiovascular responses and avoids the potential overestimation inherent in formula-based predictions. Particularly, since cycling primarily engages the lower body musculature, which can result in lower HR_max_ values compared to whole body activities. Consequently, the upper limit of 85% HR_max_ in our study does not equate to vigorous intensity in the context of our specific exercise modality. To address this, we have revised the 2.2.2 section to include a clear description:

“Prior to formal training, each participant underwent an incremental load cycling test to determine their individual HR_max_. Based on this, the target heart rate range for training was set at 60–85% of each participant's HR_max_ [42].”

We hope this clarification adequately addresses your concern.

Comment 10

- Lines 166-167. A reference should be provided for the use of HR and HRV for the quantification of the internal load.

Response:

Thank you for pointing out that a reference should be provided for the use of heart rate recovery (HRR) and heart rate variability (HRV) in quantifying internal training load. In the revised manuscript, we have added the appropriate citations in the 2.2.3 section to support this approach. The references are as follows:

“35. Mishica C, Kyröläinen H, Hynynen E, Nummela A, Holmberg H-C, Linnamo V. Relationships between Heart Rate Variability, Sleep Duration, Cortisol and Physical Training in Young Athletes. J Sports Sci Med. 2021;20(4):778-88.

45.	Da Silva DF, Ferraro ZM, Adamo KB, Machado FA. Endurance Running Training Individually Guided by HRV in Untrained Women. Journal of strength and conditioning research. 2019;33(3):736-46.

46.	Granero-Gallegos A, González-Quílez A, Plews D, Carrasco-Poyatos M. HRV-Based Training for Improving VO 2max in Endurance Athletes. A Systematic Review with Meta-Analysis. International journal of environmental research and public health. 2020;17(21):7999. doi: 10.3390/ijerph17217999.

47.	Roy S, Singh N, Gupta R. Heart rate variability in different intensities of static and dynamic exercises graded using similar heart rate. Sport Sci Health. 2020;16:669-75.

48.	Cosmin G. Heart rate variability and heart rate recovery as prognostic factors. Clujul medical. 2015;88(3):304-9.

49.	Hwang I-S, Noh Y-S, Jeong I-C, Yoon H-R. Optimized exercise load control system based on Heart Rate Variability. Biomedical Engineering Letters. 2011;1(4):232-8.

50.	Daanen HA, Lamberts RP, Kallen VL, Jin A, Van Meeteren NL. A systematic review on heart-rate recovery to monitor changes in training status in athletes. International journal of sports physiology and performance. 2012;7(3):251-60. Epub 2012/02/24. doi: 10.1123/ijspp.7.3.251.

51.	P LR, S M, J B, I LM. Adapting workload improves the measurement of heart rate recovery. International journal of sports medicine. 2011;32(9):698-702.

52.	Bilsel T, Terzi S, Akbulut T, Sayar N, Hobikoglu G, Yesilcimen K. Abnormal heart rate recovery immediately after cardiopulmonary exercise testing in heart failure patients. International heart journal. 2006;47(3):431-40. doi: 10.1536/ihj.47.431.

53.	Ganzevles SPM, de Haan A, Beek PJ, Daanen HAM, Truijens MJ. Heart-Rate Recovery After Warm-up in Swimming: A Useful Predictor of Training Heart-Rate Response? International journal of sports physiology and performance. 2017;12(6):742-8. doi: 10.1123/ijspp.2016-0144.

54.	Le Meur Y, Buchheit M, Aubry A, Coutts AJ, Hausswirth C. Assessing Overreaching With Heart-Rate Recovery: What Is the Minimal Exercise Intensity Required? International journal of sports physiology and performance. 2017;12(4):569-73. doi: 10.1123/ijspp.2015-0675.

55.	Villelabeitia-Jaureguizar K, Vicente-Campos D, Senen AB, Jiménez VH, Garrido-Lestache MEB, Chicharro JL. Effects of high-intensity interval versus continuous exercise training on post-exercise heart rate recovery in coronary heart-disease patients. Int J Cardiol. 2017;244:17-23. doi: 10.1016/j.ijcard.2017.06.067.”

A more detailed discussion of the rationale and evidence for using HRR and HRV as indicators of internal load is also provided in the Discussion section.

We appreciate your helpful feedback, which has improved the clarity and completeness of our manuscript.

Comment 11

- Lines 196-198. It is not clear how the parameter estimation was performed and which output were included in the cost function.

Response:

Thank you for your feedback regarding the clarification of the parameter estimation process and the outputs included in the cost function. We recognize that our initial submission did not clearly explain these aspects and have now enhanced the relevant section with more comprehensive details.

The revised Section 2.3.1 reads as follows:

“Using the first 80% of training day data from each subject as the model learning database, model fitting and parameter estimation were performed using MATLAB 2021a (MathWorks, USA). Three internal load indicators (ΔHRR1、HRr%、TLHRV) were used to calculate the output indicators of the model for fitting separately, as detailed in Section 2.2.3. These output indicators were then used in the cost function. The parameters were estimated by minimizing the sum of squared errors (SSE) between model-predicted values and observed values, using nonlinear least-squares minimization via the Trust-Region algorithm within the Curve Fitting Toolbox. The optimization process utilized the toolbox's default configuration, including automatic initial parameter generation and standard convergence criteria. Initial conditions were handled by treating the integration constants *C_1_* and *C_2_* as adjustable parameters, which were estimated together with the other model coefficients (*a*, *τ_a_*, *K_a_*, *f*, *τ_f_*, *K_f_*). The fitted model surfaces were visualized as three-dimensional plots. The goodness of fit was evaluated using the coefficient of determination (R^2^), SSE, and root mean square error (RMSE).”

We sincerely appreciate your feedback and believe these revisions have significantly improved the clarity and reproducibility of our methodology.

Comment 12

- Line 215. Which are the training data the Authors are referring to? HR data?

Response:

Thank you for requesting clarification on the term “training data.” In the revised manuscript we have rephrased the sentence in the Results section (3.1 section) as follows:

“Training data comprised paired external load and internal load measurements from each session, with 28–42 days of records per participant and a total of 420 paired data from 13 subjects.”

This wording clearly indicates that these data are the external load (the bicycle speed and resistance level) and internal load (HRV and HRR related indicator values) indicators, which are detailed in the Methods Section (Section 2.2.3). We trust this resolves your concern.

Thank you again for your comment.

Comment 13

- Lines 264-265. On the basis of which results is possible to infer this observation?

Response:

Thank you for raising this question. We recognized that the original statement might have appeared overly absolute. Additionally, we have updated new statistical tests to support this conclusion. The specific modifications are as follows.

Firstly, to avoid potential misinterpretations, we have revised the sentence to better reflect the results of our study in the first paragraph of Discussion.

“The fitting effect and predictive ability of the optimized fitting model are generally better than those of the original model, though individual variation exist.” This revision acknowledges the overall trend observed in the data while also being sensitive to the individual differences.

In addition, we have also added statistical tests comparing the performance of the optimized model with that of the optimized model. The specific methods are presented in Section 2.3.2:

“To further evaluate the performance of the fitted model, the above indicators were compared with the same indicators’ values obtained from the FFM original model before optimization [20, 38]. The normality of differences was assessed using the Shapiro-Wilk test. Paired t-tests (for normally distributed differences) or Wilcoxon tests (for non-normally distributed differences) were then performed, with Bonferroni correction for multiple comparisons. All analyses were conducted using MATLAB 2021a (MathWorks, USA) at a significance level of *p*<0.05.”

The results of these statistical tests can also provide more objective support for our conclusion, as mentioned in Section 3.2:

“The fitting effect of the original model is significantly worse than that of the optimized fitting model in this study. Statistical analysis revealed significant increases in R^2^ for ΔHRR1 (Bonferroni-corrected *p*=0.030) and HRr% (Bonferroni-corrected *p*=0.021). Moreover, most fitting indicators showed consistent positive trends, as presented in Fig. 2. The SSE, RMSE, and R^2^ values obtained through fitting with different output indicators are also shown in Fig 2. Specific values for each subject are provided in S5-S7 Tables.”

We have also dedicated the fourth and fifth paragraphs of the discussion section to an in-depth exploration of the potential causes of the individual variations. We believe these revisions provide a more balanced and comprehensive perspective on our findings.

Thank you again for your careful review and insightful feedback.

# To editorial office:

Comment 1

Please ensure that your manuscript meets PLOS ONE's style requirements, including those for file naming.

Response:

Thank you for your reminder. We have checked the manuscript format against the two PLOS ONE formatting sample PDFs you provided to ensure compliance. Additionally, we have downloaded the “PLoS.ens” reference format from the PLOS official website and applied it to standardize the references using EndNote software.

It is worth mentioning that changes made to the references through EndNote do not seem to be captured by the track changes mode, so the reference section still appears in black font and does not highlight the modifications.

Please feel free to contact us if there are any issues.

Comment 2

Thank you for stating the following financial disclosure:

This work was supported by the National Key R&D Program of China Foundation (2018YFC2000600); and the Fundamental Research Funds for Central Universities (2024016).

Please state what role the funders took in the study. If the funders had no role, please state: "The funders had no role in study design, data collection and analysis, decision to publish, or preparation of the manuscript."

If this statement is not correct you must amend it as needed.

Please include this amended Role of Funder statement in your cover letter; we will change the online submission form on your behalf.

Response:

Thank you for your guidance regarding the financial disclosure. We have included the following statement in the second paragraph of the cover letter:

“This work was supported by the National Key R&D Program of China Foundation (2018YFC2000600) and the Fundamental Research Funds for Central Universities (2024016). The funders had no role in study design, data collection and analysis, decision to publish, or preparation of the manuscript.”

Thank you for your kind work.

Comment 3

Your ethics statement should only appear in the Methods section of your manuscript. If your ethics statement is written in any section besides the Methods, please delete it from any other section.

Response:

Thank you for your feedback on the ethics statement. In response to your suggestion, we have deleted the separate ethics statement from the end of the manuscript. The ethics statement has been retained in subsection 2.2.1 of the Methods section.

Comment 4

In the online submission form, you indicated that more detailed datasets generated during the current study are not publicly available due to subsequent experimental projects are still ongoing but are available from the corresponding author upon reasonable request.

All PLOS journals now require all data underlying the findings described in their manuscript to be freely available to other researchers, either a. In a public repository, b. Within the manuscript itself, or c. Uploaded as supplementary information.

This policy applies to all data except where public deposition would breach compliance with the protocol approved by your research ethics board. If your data cannot be made publicly available for ethical or legal reasons (e.g., public availability would compromise patient privacy), please explain your reasons on resubmission and your exemption request will be escalated for approval.

Response:

Thank you for your attention. We fully respect the journal's data availability policy.

In response to the journal's requirements, we have uploaded all data underlying the findings described in our manuscript as supplementary information (S1_File). We have also referenced this data within the text of our manuscript in Section 3.1, where we state: “The detailed training data for each training session of all participants can be found in S1 File.”

Thank you for your understanding and support.

Comment 5

We notice that your supplementary tables are included in the manuscript file. Please remove them and upload them with the file type 'Supporting Information'. Please ensure that each Supporting Information file has a legend listed in the manuscript after the references list.

Response:

Thank you for your reminder. We have removed the supplementary tables from the manuscript file as suggested, while retaining the legends. The full content of the supplementary tables is now provided in the Supporting Information files. Please let us know if any further adjustments are needed.

Yours sincerely,

Wenxing Wang, Yuanhui Zhao, Xiao Hou, Wenlang Yu and Hong Ren
